# Routine lung ultrasound to detect postoperative pulmonary complications following major abdominal surgery: a prospective observational feasibility study

**DOI:** 10.1186/s13089-019-0135-6

**Published:** 2019-09-16

**Authors:** H. R. Touw, A. E. Schuitemaker, F. Daams, D. L. van der Peet, E. M. Bronkhorst, P. Schober, C. Boer, P. R. Tuinman

**Affiliations:** 1 Department of Anaesthesiology, Amsterdam UMC, Vrije Universiteit Amsterdam, Amsterdam Cardiovascular Sciences, De Boelelaan 1117, 1081 HV Amsterdam, The Netherlands; 2 Department of Intensive Care Medicine, Amsterdam UMC, Vrije Universiteit Amsterdam, Amsterdam Cardiovascular Sciences, De Boelelaan 1117, 1081 HV Amsterdam, The Netherlands; 3 Department of Surgery, Amsterdam UMC, Vrije Universiteit Amsterdam, Amsterdam Cardiovascular Sciences, De Boelelaan 1117, 1081 HV Amsterdam, The Netherlands; 4 Department of Intensive Care Medicine, Radboud University Medical Center, Geert Grooteplein Zuid 10, 6525 GA Nijmegen, The Netherlands; 5 Department of Health Evidence, Radboud University Medical Center, Geert Grooteplein Zuid 10, 6525 GA Nijmegen, The Netherlands

**Keywords:** Surgical procedures, Abdominal surgery, Postoperative complications, Chest X-ray and lung ultrasound

## Abstract

**Background:**

Postoperative pulmonary complications after major abdominal surgery are associated with adverse outcome. The diagnostic accuracy of chest X-rays (CXR) to detect pulmonary disorders is limited. Alternatively, lung ultrasound (LUS) is an established evidence-based point-of-care diagnostic modality which outperforms CXR in critical care. However, its feasibility and diagnostic ability for postoperative pulmonary complications following abdominal surgery are unknown. In this prospective observational feasibility study, we included consecutive patients undergoing major abdominal surgery with an intermediate or high risk developing postoperative pulmonary complications according to the Assess Respiratory risk In Surgical patients in CATalonia (ARISCAT) score. LUS was routinely performed on postoperative days 0–3 by a researcher blinded for CXR or other clinical findings. Then, reports were drawn up for LUS concerning feasibility and detection rates of postoperative pulmonary complications. CXRs were performed on demand according to daily clinical practice. Subsequently, we compared LUS and CXR findings.

**Results:**

A total of 98 consecutive patients with an ARISCAT score of 41 (34–49) were included in the study. LUS was feasible in all patients. In 94 (95%) patients, LUS detected one or more postoperative pulmonary complications during the first four postoperative days. On day 0, LUS detected 31 out of 43 patients (72.1%) with one or more postoperative pulmonary complications, compared to 13 out of 36 patients (36.1%) with 1 or more postoperative pulmonary complications detected with CXR RR 2.0 (95 CI [1.24–3.20]) (*p* = 0.004). The number of discordant observations between both modalities was high for atelectasis 23 (43%) and pleural effusion 29 (54%), but not for pneumothorax, respiratory infection and pulmonary edema 8 (15%), 3 (5%), and 5 (9%), respectively.

**Conclusions:**

This study shows that LUS is highly feasible and frequently detects postoperative pulmonary complications after major abdominal surgery. Discordant observations in atelectasis and pleural effusions for LUS and CXR can be explained by a superior diagnostic ability of LUS in detecting these conditions. The effects of LUS as primary imaging modality on patient outcome should be evaluated in future studies.

## Background

There is increasing interest in early detection of postoperative pulmonary complications (PPCs) to reduce patient morbidity and mortality [1]. Furthermore, in patients unexpectedly admitted to the intensive care unit (ICU), insufficient diagnostic imaging was often performed on the general ward and inadequate treatments were initiated [2]. Early PPC detection would enable physicians to start treatment in time and, therefore, prevent its negative impact on patient outcome.

Respiratory failure following anaesthesia and surgery typically starts with atelectasis, due to diaphragmatic dysfunction, inability to clear secretions and immobility. Chest auscultation and chest X-ray (CXR) are frequently used as diagnostic modalities to detect PPCs, but have limited diagnostic accuracy [3–6]. Moreover, ideally, radiography requires an upright position of the patient, and multiple concomitant lung abnormalities further complicate the two-dimensional interpretation [7–9]. Computed tomography (CT) is the gold standard for pulmonary pathology but requires significant ionizing radiation and the need for transfers within the hospital, which is not without risk [3].

Alternatively, lung ultrasound (LUS) is a quick, point-of-care and radiation free technique. It has an excellent diagnostic accuracy for atelectasis, pulmonary effusions, pulmonary edema, and/or pneumonia in critically ill patients compared to the gold standard CT [3, 4, 6]. Therefore, LUS is well established in these pathologies, and practical approaches to perform LUS have been described extensively elsewhere [10, 11]. We previously showed that, in cardiothoracic surgery patients, LUS detected more (clinically relevant) PPCs compared to CXR and at an earlier time point [12]. Following major abdominal surgery, the feasibility could be limited by dressings, chest drains, or subcutaneous emphysema.

Daily routine CXR has been largely abandoned in ICU and cardiothoracic patients due to its limitations, costs, and radiation burden. On demand, CXR performance has led to a significant decrease in the number of CXRs without any increase in adverse events in ICU patients [13]. Studies comparing on-demand strategies with routine strategies are lacking in patients after major abdominal surgery. However, performing CXR on demand does not facilitate early detection of postoperative pulmonary complications. Potentially, routine LUS can detect PPCs early and with higher accuracy to improve perioperative management in major abdominal surgery patients [4, 10, 14].

The primary objective of this pilot study was to test the feasibility of routine LUS following major abdominal surgery and to report the detected PPCs with LUS in these postoperative patients. Subsequently, PPC detection rates for LUS and CXR were compared.

## Methods

We conducted this prospective, observational feasibility study in the Amsterdam UMC—VU University Medical Center Amsterdam (VUmc Amsterdam, The Netherlands), a tertiary hospital. The local Human Subjects Committee of the VUmc approved the study (METc 16/128), and written informed consent was obtained from all participants. Patient inclusion started June 2016 and ended March 2017. Patients were included on the general ward. The study included consecutive adult patients (age ≥ 18 years) scheduled for elective major abdominal (e.g., gastrointestinal, vascular, or renal) surgery with an intermediate or high risk for the development of postoperative pulmonary complications according to the Assess Respiratory risk In Surgical patients in CATalonia (ARISCAT) risk score ≥ 26 (https://www.mdcalc.com/ariscat-score-postoperative-pulmonary-complications) [15, 16]. Exclusion criteria were trauma or emergency surgery.

### Patient characteristics

Patient characteristics were retrieved from the patient data management system and included age, gender, weight, length and body mass index (BMI), American Society of Anesthesiology (ASA) classification, the ARISCAT score, co morbidities, alcohol use, and smoker status. Perioperative parameters were also retrieved and included type of surgery, ICU referral, and total in-hospital length of stay. Patient and laboratory data included the following: arterial oxygen saturation, mode of ventilation, O_2_ supplementation, sputum cultures, temperature, and leukocyte count.

### Chest X-ray

CXR was performed according to standard clinical practice (e.g., after placement of chest tube or central venous line placement) and when demanded by the treating physician for clinical reasons. Anteroposterior bedside CXRs were obtained using a DRX-Revolution mobile X-ray unit (Carestream Health, Inc. © Toronto, Canada). CXR findings, assessed by a radiologist blinded to the LUS findings, were retrospectively retrieved from the patient data management system (PDMS). The Nomenclature Committee of the Fleischner Society recommended terminology was used to describe pathological entities according to the diagnostic criteria for bedside CXR [17, 18].

### Lung ultrasound

LUS was routinely performed by a trained member of the research team (*n* = 3), which consisted of two dedicated investigators and one ICU fellow. LUS was performed upon admission after surgery on postoperative day (POD) 0, and on all consecutive PODs 1–3. Daily LUS was intended, but ultimately could not be performed in a large proportion of the time because of limited researcher availability, e.g., admission after working hours and follow-up days in the weekends. All ultrasonographers were trained according to The Netherlands Society of Intensive Care Programme for Intensive Care Ultrasound (NVIC ICARUS) training course (https://www.nvic.nl). The ultrasonographer was blinded to clinical details and CXR findings, and did not contribute to the diagnostic and treatment strategy of the patient. We used a CX50 ultrasound machine (Koninklijke Philips NV^®^, Eindhoven, The Netherlands) with both the cardiac phased array (1-5 MHz) and the linear vascular probe (> 10 MHz). The ultrasonographer could choose a particular probe for a particular view in a particular patient, according to individual preference. Lung sliding was determined using the vascular probe. LUS views were obtained according to the BLUE protocol [13, 19].

**Fig. 1:**
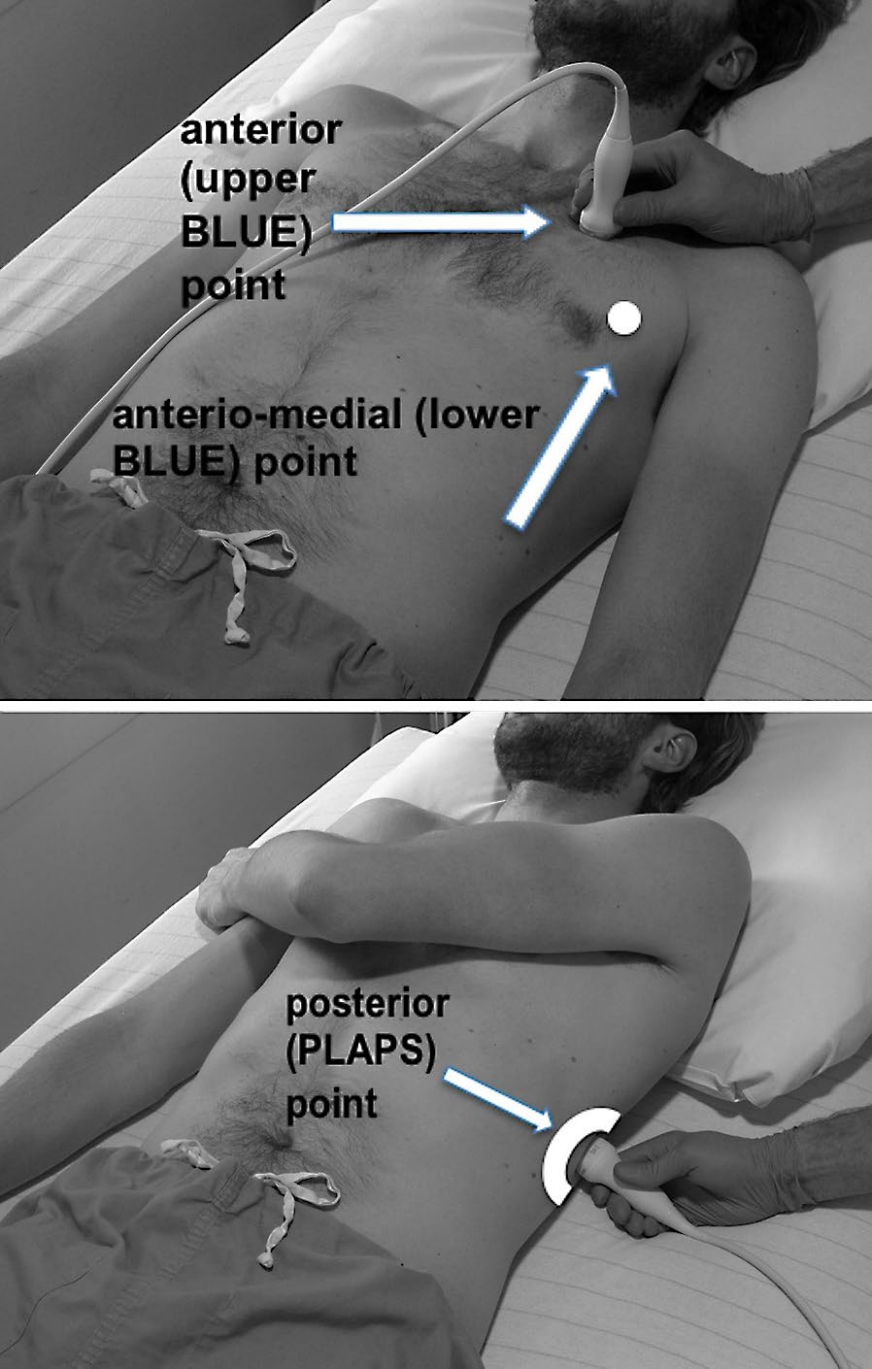
BLUE and PLAPS points. Anterior (upper BLUE) point. Anteromedial (lower BLUE) point. Posterior (PLAPS) point

**Fig. 2:**
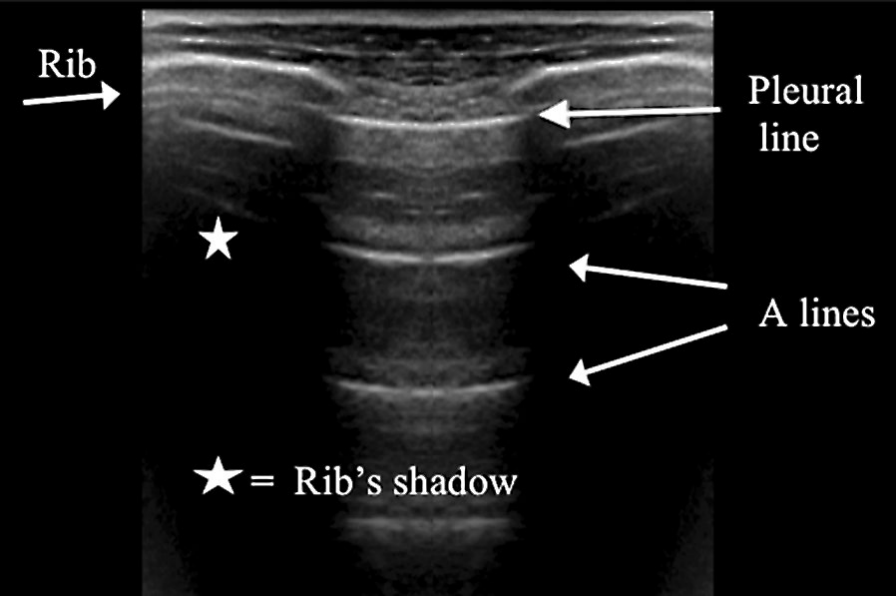
A profile according to the BLUE protocol showing multiple A lines

**Fig. 3:**
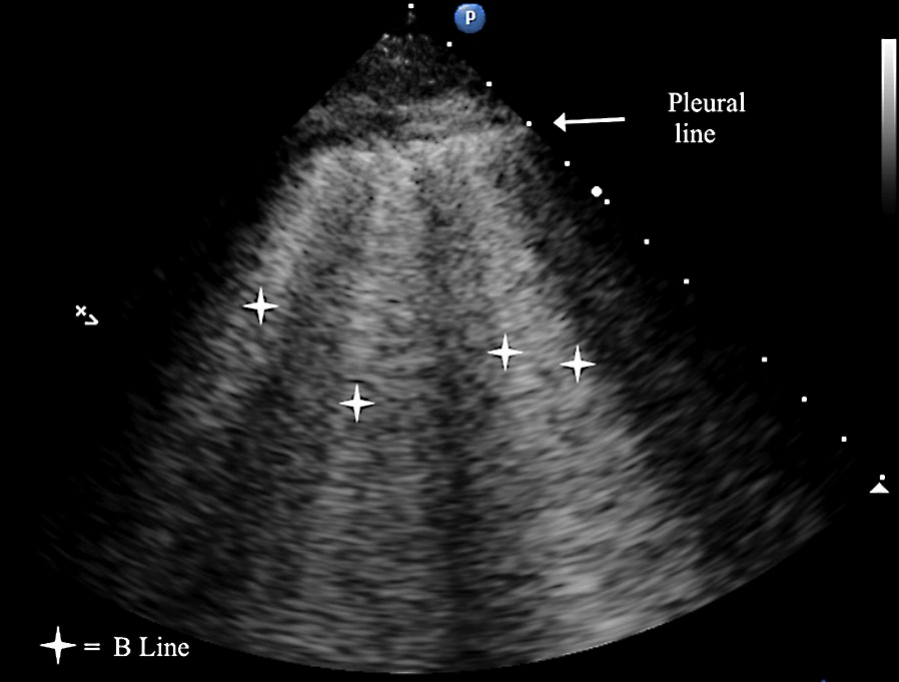
B profile according to the BLUE protocol showing multiple B lines

**Fig. 4:**
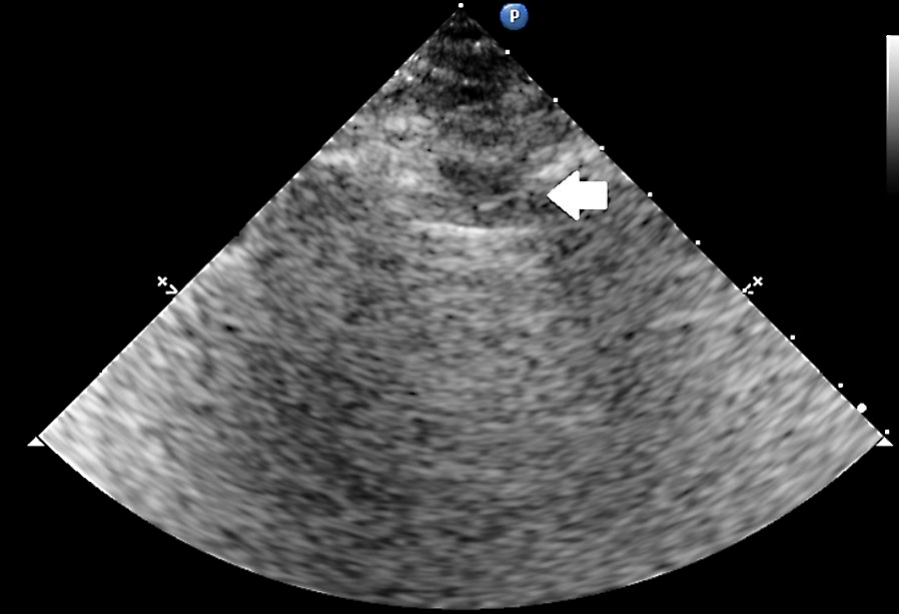
C profile according to the BLUE protocol showing a C line. Arrow indicates a hypoechoic subpleural focal image generated by consolidated lung tissue

**Fig. 5:**
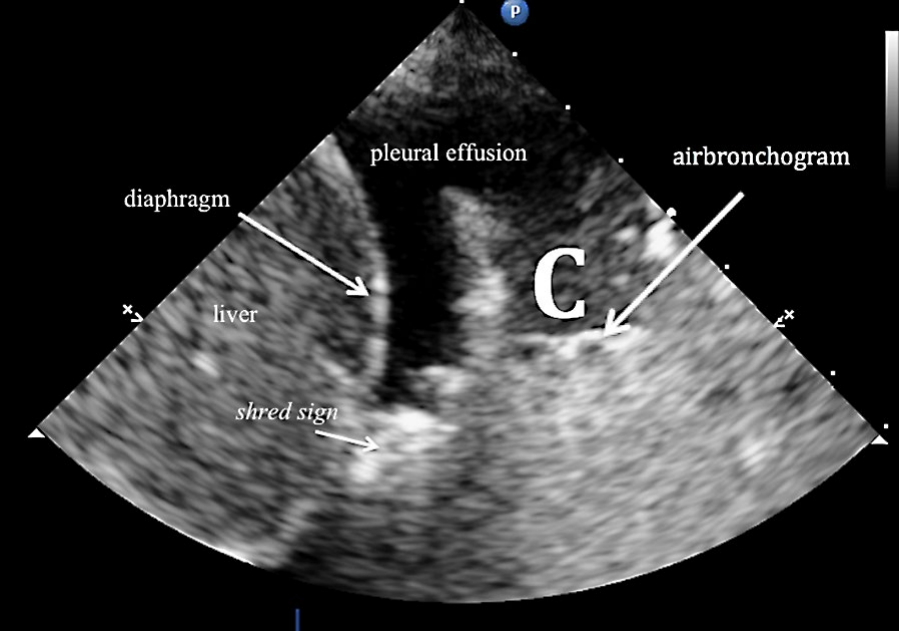
Posterolateral alveolar and/or pleural syndrome

LUS was performed according the BLUE protocol and adjusted for the postoperative setting [12]. BLUE profile for each BLUE point (Fig. 1) and BLUE profile per hemithorax (BLUE 1 and 2) was determined as follows: A; B; A′; B′; or C profile. A profile means predominantly A lines (Fig. 2). B profile means predominantly multiple (> 2) anterior diffuse B lines indicating interstitial syndrome (Fig. 3). A′ or B′ means the corresponding BLUE profile with absence of, or abolished lung sliding. C profile means anterior alveolar consolidation (Fig. 4). Furthermore, we determined the postero-lateral alveolar and/or pleural syndrome (PLAPS) (Figs. 1, 5) at the lateral sub-posterior sides of the chest and scored them as positive or negative. When positive, consolidation and/or pleural effusion were scored separately and the diagnosis of atelectasis was added to the flowchart. The final conclusion was made according to the bedside LUS protocol in cardiothoracic patients, as published in the previous research [12]. For differentiation between pneumonia and atelectasis, the ultrasonographer was also allowed to look for fever, leukocyte count, and C-reactive protein (CRP) in the PDMS (data also available to the radiologist).

### Postoperative pulmonary complications

The incidence of PPCs detected with CXR was retrieved by a research team member using the PDMS. PPCs were scored according to reported radiology findings of on-demand CXR studies ordered by the treating physician at the ward. PPCs were scored as previously defined by Canet et al.: respiratory infection defined as treatment with antibiotics for a suspected respiratory infection and at least one of the following criteria: new or changed sputum, new or changed lung opacities, fever, leukocyte count > 12,000/mm^3^; pleural effusion defined by blunting of the costophrenic angle, loss of the sharp silhouette of the ipsilateral hemidiaphragm in upright position, evidence of displacement of adjacent anatomical structures, or (in supine position) a hazy opacity in one hemithorax with preserved vascular shadows; atelectasis defined by lung opacification with shift of the mediastinum, hilum, or hemidiaphragm towards the affected area and compensatory over-inflation in the adjacent non-atelectatic lung; and pneumothorax defined by the air in the pleural space with no vascular bed surrounding the visceral pleura, all demonstrated by the CXR [15, 16].

### Clinically relevant postoperative pulmonary complications

Clinically relevant PPCs, defined as a PPC that required treatment, as judged by the treating physician, were also reported [12]. We considered the following initiated treatments for scoring clinically relevant PPCs: re-intubation; ICU admission; bronchoscopy; extra bronchodilator therapy; thoracic drain placement; and the use of diuretics and/or antibiotics. Scores of clinically relevant PPCs were retrospectively retrieved from the PDMS according to initiated treatment by the treating physicians for PPCs at the ward. For example: a found pneumothorax which did not require drainage was counted as a PPC but not as a crPPC. The physician who diagnosed clinically relevant PPCs took the following into account: physical examination, conventional monitoring, laboratory results, CXR, and/or CT scan results when available. This reflected daily clinical practice. The treating physicians were blinded to LUS findings.

### Statistical analysis

We included as many consecutive patients as possible in the study period. We planned to include more than 90 patients to detect around 30 PPCs based on incidence rates of PPCs after surgery [16]. Results of this study will be used to plan a larger study on the effects of LUS on patient outcome after major abdominal surgery. We performed statistical data analyses using SPSS statistical software package version 22.0 (IBM, New York, NY, USA). Feasibility was reported as percentage of patients in which LUS was possible. Detection rates of PPCs during the study period were reported as frequencies. A PPC could only develop once. For example, if a pleural effusion was detected on POD1, it could not be scored again on POD2. However, a patient could develop more than one PPC during the study period and during 1 day. In addition, detection rates were compared according to both imaging modalities. Furthermore, an aggregated analysis was performed to compare the detection of PPCs in patients who had both; CXR and LUS were performed the same day during the study period. Relative risk was calculated comparing PPC detection with LUS and CXR. Discordant observations were compared for PPCs detected with LUS and CXR.

## Results

115 patients were considered eligible for inclusion. 110 patients signed informed consent, 12 were excluded because of postoperative correction of initial ARISCAT score (*n* = 7) or cancelling informed consent (*n* = 5). Overall, 98 consecutive patients were included in the study. Table 1 shows the patient characteristics of included patients. One patient died within the 30 day follow-up period.

**Table 1:** Perioperative patient characteristics

Patient characteristics	Values
*N*	98
Males/females (*n*)	59/39
Age (years)	64 ± 11.7
Body mass index (kg/m^2^)	26.1 ± 4.6
Hypertension	39 (39.8)
COPD	11 (11.2)
Oncology	79 (81.4)
Median ASA physical status	2 [2, 3]
Median ARISCAT score	41 [34-49]
Types of surgery, *n* (%)	
Esophageal	14 (14.3)
Gastric	9 (9.2)
Hepatic	19 (19.4)
Pancreatic	12 (12.2)
Vascular	6 (6.1)
Renal	16 (16.3)
Other gastrointestinal	22 (22.4)

Data represent mean ± standard deviation, median with [interquartile range] or number of cases (percentage)

*ASA* American Society of Anesthesiologists, *ARISCAT* Assess Respiratory risk In Surgical patients in CATalonia

### Routine lung ultrasound: feasibility and incidence of PPCs

In 100% of patients, LUS was feasible. In total, routine LUS detected in 94 out of 98 patients (96%) one or more PPCs in the study period. Table 2 shows the incidence rates of newly detected PPCs with LUS.

**Table 2:** Incidence rates per day of newly detected postoperative pulmonary complications with point-of-care lung ultrasound after major abdominal surgery

Day	Number of patients in which LUS is performed (*N*)	Patients with ≥ 1 PPC [*N* (%)]	Respiratory infection	Pneumothorax	Pulmonary edema	Atelectasis	Pleural effusion
POD 0	43	31 (72%)	2	2	0	29	10
POD 1	92	64 (70%)	0	4	2	55	31
POD 2	87	21 (24%)	4	0	1	9	9
POD 3	56	8 (14%)	1	0	2	1	5

Patients can develop multiple PPCs. Total amount of PPCs can be higher than the number of patients

*LUS* lung ultrasound, *PPC* postoperative pulmonary complication, *POD* postoperative day

### Lung ultrasound compared with chest X-ray

CXR examinations performed and incidence rates of PPCs detected are shown in Table 3. 36 out of 98 patients (37%) with one or more PPCs were detected with on-demand CXR in the study period. On POD 0, LUS detected 31 out of 43 patients (72.1%) with one or more PPCs, compared to 13 out of 36 patients (36.1%) with one or more PPCs detected with CXR (*p* = 0.004) RR 2.0 (95% CI [1.24–3.20]). LUS and CXR mainly identified atelectasis and pleural effusion, as shown in Tables 2 and 3. However, the number of discordant observations between CXR and LUS for atelectasis and pleural effusion was much higher: 23 and 29 (43% and 54%), respectively. (Table 4) For pneumothorax, respiratory infection and pulmonary edema the number of discordant pairs were 8, 3, and 5 (15%, 5%, and 9%), respectively.

**Table 3:** Incidence rates of newly detected postoperative pulmonary complications with ‘on-demand’ chest X-ray after major abdominal surgery according to daily clinical practice

Day	Number of patients in which CXR is performed (on demand)	Patients with *N* ≥ 1 PPC [*N* (%)]	Respiratory infection	Pneumothorax	Pulmonary edema	Atelectasis	Pleural effusion
POD 0	36	13 (36%)	0	2	3	9	4
POD 1	19	13 (72%)	0	4	3	9	6
POD 2	13	7 (54%)	0	2	0	3	5
POD 3	9	3 (33%)	0	1	1	1	3

Patients can develop multiple PPCs. Total amount of PPCs can be higher than the number of patients

*CXR* chest X-ray, *PPC* postoperative pulmonary complication, *POD* postoperative day

**Table 4:** Discordant pairs of newly detected atelectasis and pleural effusion for lung ultrasound and chest X-ray when concomitantly performed (*n* = 54) between days 0 and 4 in 98 patients after abdominal surgery

Atelectasis	LUS			Pleural Effusion	LUS		
	No	Yes	Total		No	Yes	Total
CRX				CRX			
No	10	17	27	No	15	15	30
Yes	6	11	17	Yes	14	10	24
Total	16	28	54	Total	29	25	54

### The incidence of clinically relevant postoperative pulmonary complications

In total, 13 patients (13%) developed a clinically relevant PPC. Six patients were readmitted to the ICU and 1 was re-intubated for respiratory failure. The incidence of new clinically relevant PPCs during the study period POD 0–4 is shown in Table 5. The incidence of new clinically relevant PPCs increased over the first 4 days, with a maximum of 10 PPCs on POD 3. For 8 patients, the incidence of respiratory infection could be followed up to 30 days postoperatively. LUS detected consolidations in 7 out of 8 patients treated for respiratory infection and CXR detected consolidations in 5 out of 8 patients during the study period.

**Table 5:** Incidence of newly detected clinically relevant postoperative pulmonary complications on days 0, 1, 2, 3, and 4–30 in 98 patients after abdominal surgery

Clinically relevant PPCs	Day 0	Day 1	Day 2	Day 3	Day 4-30
Pneumonia	0	0	0	3	5
Pneumothorax	0	0	0	1	0
Pulmonary edema	0	1	3	3	0
Atelectasis	0	0	2	1	0
Pleural effusion	0	2	1	2	2

Data represent number of cases (percentage)

*PPCs* postoperative pulmonary complications

## Discussion

This pilot study demonstrates that routine LUS is highly feasible and frequently detects PPCs in patients following major abdominal surgery. In addition, LUS detected more PPCs when compared to CXR when both modalities were performed on POD 0. Importantly, the discordant observations for LUS and CXR were highest for atelectasis and pleural effusion.

We confirm that LUS is highly feasible in postoperative patients [12, 20, 21]. Goudy et al. found that LUS was feasible in 97% of the hemi thoraxes in 252 ultrasound examinations after cardiothoracic surgery, despite the presence of chest drainage tubes or applied dressings [21]. To the best of our knowledge, we are the first to report high feasibility following major abdominal surgery. Following major abdominal surgery, dressings, chest drains, or subcutaneous emphysema or limited patient mobility could have prohibited the feasibility in this setting.

We cannot compare our results with the previous research, because this is the first study to report PPC rates detected by LUS following major abdominal surgery. The rates found with LUS are high in comparison with PPC rates previously reported with other diagnostic modalities [15]. However, in the cardiothoracic surgery population, it was previously reported that the rate of PPCs detected with LUS was up to 100% [12, 22]. Detection of PPCs with CXR in our study (37%) is in line with preoperative risk scores [16]. In addition, Mazo et al. recently showed incidence rates of PPCs ranging from 38 to 50% in a large validation study in Western Europe in a comparable study population [16].

We found that LUS and CXR, when performed at the same time, identified pleural effusion and atelectasis in different patients (discordant pairs) following major abdominal surgery. These discordant pairs were detected in up to 50% of the patients who had both imaging modalities performed on the same day. This difference can be explained by a higher sensitivity and specificity of LUS in detecting pleural effusion and atelectasis compared to CXR [3, 6, 20]. In a recent systematic review and meta-analysis, LUS had an overall sensitivity of 95% (92–96%) and specificity of 94% (90–97%) compared to CT in adult critically ill patients with respiratory symptoms [6]. CXR had an overall sensitivity of 49% (40-58%) and specificity of 92% (86–95%) in the same systematic review and meta-analysis. Therefore, LUS is being considered by others as the bedside gold standard for pulmonary pathology in critically ill patients with respiratory symptoms [4, 14].

In a recent pilot study performed in cardiothoracic surgery evaluating effectiveness of LUS as the primary diagnostic imaging technique, LUS was exhaustive in up to 80% of the patients. Subsequent CXR was not required in the postoperative management of these patients to assist decision making in this study and LUS was considered effective in perioperative patient management. Furthermore, LUS allowed further discrimination of lung abnormalities mainly between atelectasis and pleural effusions. [22, 23].

Other advantages of LUS are that the time to perform LUS was significantly shorter than for CXR in the postoperative setting [12] and LUS had excellent inter-observer agreement [4, 12]. Furthermore, LUS can be used to guide central venous catheter placement and position [24], thoracentesis, and the management of fluid administration in acute circulatory failure [11]. However, if routine LUS findings are inconclusive in patients with respiratory symptoms, additional imaging is warranted; we do suggest using additional CT imaging to make a final diagnosis.

Our study has important limitations. LUS examinations could not be performed on all postoperative days. On POD 0, patients were frequently admitted after working hours, and, on POD 3, was frequently during weekends. Consequently, researchers were not always available to perform LUS. Therefore, the predictive value of detected complications to become clinically relevant PPCs could not be analysed. Another important limitation is the absence of the gold standard (computed tomography) for lung pathology to relate our LUS findings. We chose to use and adjust the most practical, succinct, but well validated and accurate LUS protocol (BLUE protocol) [14, 19]. However, detection rates could even be increased more by further quantifying B lines and to scan 8 or even 28 separate intercostal spaces instead of 4 [25]. The clinical importance of using LUS is unknown with regard to detecting PPCs that do not require therapy according to the treating physician. This should be studied in further studies. We strongly believe that not all atelectasis or pleural effusion need treatment. However, LUS findings, similar to other clinical findings, should always be seen in light of patient’s symptoms and health status. Our findings indicate to further study LUS as a primary screening tool for detecting PPCs in major abdominal surgery patients. Future studies should focus on patient outcome and cost effectiveness.

Strength of this study is that we have demonstrated that LUS is indeed feasible and frequently detects PPCs in patients following major abdominal surgery. The protocol we propose is easy to use, also for physicians newly trained in LUS. The protocol is based on limited, standardized signs, a major advantage of LUS, because the risk of wrong interpretations is thereby decreased.

Developing competence in LUS is considered straightforward. Surgeons and anaesthesiologists active in the postoperative period should consider training in LUS as part of their point-of-care ultrasound skills. Furthermore, adding this useful tool into resident training would parallel its broader use in other medicine specialties. Teaching the basics of LUS to perioperative physicians and residents would be fairly easy. For example, in nephrology, a 4-h course that includes pre-course cognitive preparation, a didactic lecture and training in image acquisition/interpretation is sufficient to provide the learner a strong foundation in LUS. However, the course alone is not sufficient to provide competence [26]. Competence in LUS requires additional bedside scanning of patients under the supervision of capable faculty as outlined before [10].

## Conclusions

This pilot study shows that LUS is highly feasible and detects PPCs frequently in patients after major abdominal surgery. LUS findings should be evaluated in combination with all available clinical data when considering initiating treatment. Closely monitoring patients and detecting PPCs with LUS could create a window of opportunity to limit the impact of PPCs on patient outcome. This needs to be studied in future trials.

## Abbreviations

ARISCAT score: Assess Respiratory risk In Surgical patients in CATalonia score
ASA: American Society of Anesthesiology
BMI: body mass index
BLUE protocol: bedside lung ultrasound in emergency protocol
CT: computed tomography
CRP: C-reactive protein
CPAP: continuous positive airway pressure
CXR: chest X-ray
ICU: intensive care unit
LUS: lung ultrasound
PDMS: patient data management system
PEEP: positive end-expiratory pressure
PLAPS: postero-lateral alveolar and/or pleural syndrome
POD: postoperative day
PPCs: postoperative pulmonary complications
